# Dual Tasking Affects the Outcomes of Instrumented Timed up and Go, Sit-to-Stand, Balance, and 10-Meter Walk Tests in Stroke Survivors

**DOI:** 10.3390/s24102996

**Published:** 2024-05-09

**Authors:** Masoud Abdollahi, Pranav Madhav Kuber, Ehsan Rashedi

**Affiliations:** Industrial and Systems Engineering Department, Rochester Institute of Technology, Rochester, NY 14623, USA; ma8489@rit.edu (M.A.); pmk2015@rit.edu (P.M.K.)

**Keywords:** stroke, neurological disorders, movement analysis, kinematics, rehabilitation

## Abstract

Stroke can impair mobility, with deficits more pronounced while simultaneously performing multiple activities. In this study, common clinical tests were instrumented with wearable motion sensors to study motor–cognitive interference effects in stroke survivors (SS). A total of 21 SS and 20 healthy controls performed the Timed Up and Go (TUG), Sit-to-Stand (STS), balance, and 10-Meter Walk (10MWT) tests under single and dual-task (counting backward) conditions. Calculated measures included total time and gait measures for TUG, STS, and 10MWT. Balance tests for both open and closed eyes conditions were assessed using sway, measured using the linear acceleration of the thorax, pelvis, and thighs. SS exhibited poorer performance with slower TUG (16.15 s vs. 13.34 s, single-task *p* < 0.001), greater sway in the eyes open balance test (0.1 m/s^2^ vs. 0.08 m/s^2^, *p* = 0.035), and slower 10MWT (12.94 s vs. 10.98 s *p* = 0.01) compared to the controls. Dual tasking increased the TUG time (~14%, *p* < 0.001), balance thorax sway (~64%, *p* < 0.001), and 10MWT time (~17%, *p* < 0.001) in the SS group. Interaction effects were minimal, suggesting similar dual-task costs. The findings demonstrate exaggerated mobility deficits in SS during dual-task clinical testing. Dual-task assessments may be more effective in revealing impairments. Integrating cognitive challenges into evaluation can optimize the identification of fall risks and personalize interventions targeting identified cognitive–motor limitations post stroke.

## 1. Introduction

Stroke, a pervasive global health issue, stands as a leading cause of enduring disability, inflicting a range of motor impairments that include muscle weakness, vexing spasticity, coordination loss, and precarious balance deficits [1]. These post-stroke impairments cast a formidable shadow over daily life, elevating the risk of falls and curtailing functional mobility [2]. Stroke rehabilitation programs center their efforts on evaluating and addressing these mobility challenges [3,4,5]. To this end, clinicians employ standardized clinical tests to assess various aspects of mobility [6,7,8]. For instance, the Timed Up and Go test (TUG) measures transitional movements [9,10,11], the Sit to Stand test (STS) evaluates sit-to-stand transfers [12,13], and standing balance assessments scrutinize the equilibrium provided by the neuromusculoskeletal system [14,15,16]. Moreover, the 10 m Walk Test (10MWT) quantifies gait speed, shedding light on functional independence [17,18]. Regrettably, stroke survivors (SS) often exhibit significantly poorer performance on these tests compared to their healthy peers, starkly underscoring the profound impact of stroke on mobility [19,20]. With efforts toward recovery, healthcare professionals employ these assessments as indispensable tools to guide rehabilitation, striving to restore not just physical abilities but also the quality of life of stroke survivors [8].

Standardized clinical tests have been invaluable tools in evaluating mobility deficits among SS, shedding light on the challenges they face in regaining functional independence [21,22]. However, these tests, while highly informative, may fall short in fully capturing the intricate and often subtle mobility deficits that emerge in the complex, cognitively demanding scenarios of real-world situations. Recognizing this limitation, researchers and healthcare professionals have turned to the concept of dual-task testing to bridge the gap between clinical assessments and everyday challenges [23,24,25]. Dual task testing involves the integration of cognitive tasks alongside motor assessments, simulating the multitasking demands that SS encounter in their daily lives. This approach has revealed a striking phenomenon: SS frequently exhibit more pronounced dual-task costs, indicating greater performance decrements when simultaneously juggling motor and cognitive tasks, compared to their healthy counterparts [26,27,28,29]. These findings underscore the importance of considering cognitive demands in stroke rehabilitation, as many survivors report experiencing mobility difficulties precisely when engaged in cognitive tasks or multitasking [28,30].

Understanding the implications of dual-task effects in stroke rehabilitation is of importance because dual-task performance can serve as a valuable predictive indicator for concern among SS and older adults in general—the risk of falls [31,32]. Falls can have devastating consequences for individuals recovering from stroke, often leading to injuries and setbacks in their rehabilitation journey [33]. Integrating dual-task assessments, such as quantifying dual-task costs during widely used clinical tests like the TUG, holds significant promise. By performing such tests, clinicians can gain a more comprehensive understanding of the multifaceted mobility deficits experienced by SS when they are engaged in multitasking scenarios in their daily lives. This approach enables healthcare professionals to tailor rehabilitation strategies with greater precision. Furthermore, the insights derived from dual-task assessments can inform critical decisions regarding when SS are ready to resume more cognitively complex activities within their communities. This not only enhances their overall quality of life but also increases their sense of independence, a crucial aspect of stroke recovery. In essence, the incorporation of dual-task assessments into stroke rehabilitation not only reduces the risk of falls but also empowers survivors to regain their autonomy and reengage with their surroundings more confidently [21,34].

This study evaluated the effect of stroke and concurrent motor–cognitive dual tasking on common clinical tests including the TUG, STS, standing balance tests, and 10MWT. The aims were to (1) compare single and dual-task performance between SS and healthy controls (HC) on these tests and (2) examine if there is any interaction effect of stroke and dual-tasks on the general outcomes of the tests. We hypothesized that (1) SS would exhibit greater dual-task costs compared to the controls across all tests and (2) dual-task performance would better discriminate between the groups (i.e., stroke and control) than single clinical tests alone. The findings of this study could provide evidence to support the integration of dual-task testing into stroke evaluation to better identify mobility deficits. Quantifying dual-task costs during clinical testing may help clinicians recognize SS prone to falls and guide referrals for comprehensive fall risk assessments. The results could also inform the development of tailored dual-task mobility training programs targeting identified deficits in multitasking abilities. These programs may incorporate progressive multitasking practice on tasks like the TUG to improve dual-task performance and the transference of skills learned to daily life. Improved dual-task mobility could enhance community ambulation and participation in SS by reducing fall risk and building confidence in performing daily activities requiring divided attention.

## 2. Materials and Methods

### 2.1. Participants

The study comprised 41 participants and included 21 SS and 20 HC. Anthropometric measurements showed no statistically significant differences (*p* < 0.05), as detailed in Table 1. All participants provided written informed consent prior to the experiment. Ethical approval was obtained from the Institutional Review Boards (IRBs) of both the Rochester Institute of Technology and Rochester Regional Health Hospital. All participants in the stroke group were recruited based on an inclusion criterion that mandated independent walking ability over 10 m, stroke occurrence at least six months prior, and the absence of severe medical conditions significantly impacting physical performance.

### 2.2. Study Approach

Clinical tests were conducted on two distinct groups: stroke patients (i.e., the stroke group) and HCs (i.e., the control group), under two separate conditions, namely with and without cognitive loading. The battery of selected clinical tests encompassed a range of activities, including sitting and standing, stationary standing, walking, and turning. Specifically, there were five tests, comprising the TUG, STS, balance test with open/closed eyes, and the 10MWT. The choice of these particular tests was motivated by their widespread adoption within healthcare settings for the comprehensive evaluation of motor function, locomotion, and balance performance in post-stroke patients [13,18,35]. The participants underwent the five different tests in a randomly determined sequence. Furthermore, for each test, they had two chances to begin with either the dual task or single task, with the choice being randomized.

A cognitive loading task was integrated into the dual-task condition for each participant. Prior studies have implemented a variety of cognitive loading tasks, including identifying colors, subtraction, verbal analogical reasoning, spelling backward, and counting backward [21,36]. During our pilot testing, we found counting backward to be most suitable for our study. Reverse counting has been utilized in studies similar to ours to induce cognitive loading while performing clinical tests [37,38]. Specifically, this cognitive task entailed counting backward from 200, decreasing by intervals of 10, while simultaneously performing the designated functional task. In cases where the participants reached zero before completing the task, they received instructions to restart counting from 200. This happened mostly during dual-task balance tests with open/closed eyes. It is noteworthy that the participants received explicit guidance to ensure that they tried their best to ensure that their performance remains unaffected as much as possible by the numerical counting task.

#### 2.2.1. Procedure for Clinical Tests

**Figure 1:**
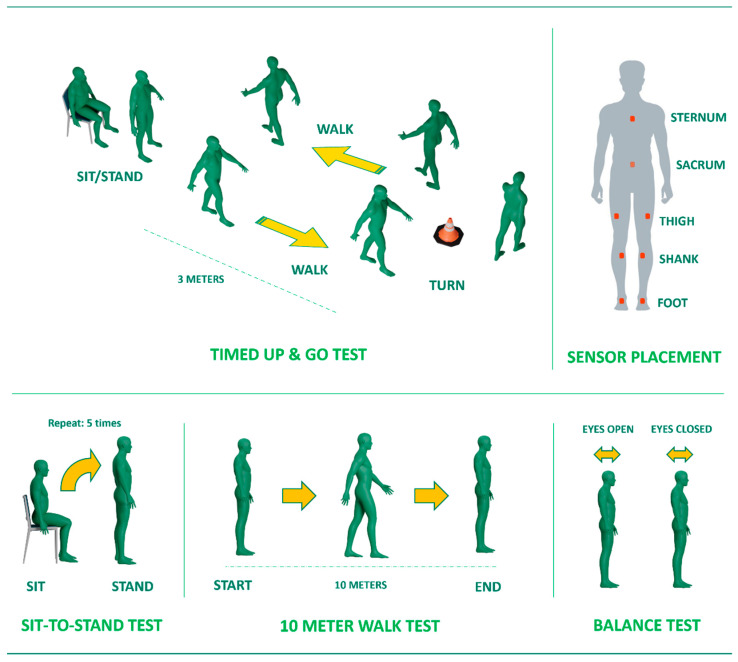
Illustration depicting the common clinical tests including (**top**-**left**) the Timed Up and Go (TUG) test, (**bottom**-**left**), the sit-to-stand (STS) test, (**bottom**-**middle**) the 10 m Walk Test (10MWT), and (**bottom**-**right**) the balance test; (**top**-**right**) placement locations of inertial sensors on the body.

This study employed five distinct clinical tests, comprising the TUG, STS, 10MWT, and balance tests with open and closed eyes, as depicted in Figure 1. The TUG test involved participants initiating the task from a seated position, followed by transitioning to a standing posture and walking to a designated cone located approximately 10 feet (3 m) from the chair’s origin. Upon reaching the cone, the participants executed a 180-degree turn around it and then returned to the chair. The conclusion of the TUG test occurred when the participants resumed a seated position. In instances where the participants reached zero before reaching the chair, they were instructed to restart counting from 200. The second test in our battery was the STS, which entailed the participants performing five trials of repetitive sitting and standing from a chair. Similarly, the 10MWT required the participants to walk in a straight line for a distance of ten meters. The final tests, the balance tests, tasked the participants with maintaining a stationary and neutral posture—hands at their sides and facing forward for 30 s. This test featured two separate conditions: one with the participants standing still while keeping their eyes open and another with their eyes closed. These five tests were chosen due to their widespread use and recognition among clinicians. Each participant performed the five tests twice—once without and once with a motor–cognitive dual-task, which required counting backward from 200 in decrements of 10 while navigating the prescribed path.

#### 2.2.2. Experimental Design and Independent Factors

This study employed a 2 × 2 mixed factorial design encompassing two distinct independent variables. The first variable, group, was utilized as a between-subjects factor, distinguishing between the SS and HI. The second variable, cognitive load (CL), served as a within-subject factor, distinguishing between the single-task and dual-task conditions. Within this experimental framework, all participants completed five clinical tests (TUG test, STS task, balance with eyes open, balance with eyes closed, and the 10MWT) under both single-task (solely performing the motor test) and dual-task (simultaneously performing the motor test while counting backward by 10 s from 200) conditions.

For three of these tests (specifically, the TUG test, STS task, and 10MWT), we focused on general outcome measures such as time taken, number of steps, and cadence. These metrics were chosen as they could be assessed by clinicians without the need for motion sensors. Our study primarily aimed to investigate how stroke and the introduction of dual-task demands influenced these general clinical outcomes, rather than delving into intricate kinematic variables. In contrast, for the balance tests, we employed inertial measurement units (IMUs) manufactured by Movella (Xsens, Enschede, The Netherlands) to collect kinematic data. This decision was made because quantifying body sway during balance tests without sensors is challenging for clinicians. Another reason for utilizing such a motion capture system was due to its portability. This motion capture system has been validated and widely used for capturing detailed body motion in out-of-lab scenarios [39,40,41]. A total of 8 IMUs were strategically positioned on various body segments, including the feet, shanks, thighs, sternum, and sacrum of each participant. These IMUs formed a predefined ‘lower-body and trunk’ configuration, as illustrated in Figure 1. Motion data from each sensor were recorded at a sampling frequency of 100 Hz using Xsens software (MVN Analyze^®^ 2021.0.1, Xsens, Enschede, The Netherlands) in synchronization with the administration of the tests.

### 2.3. Data and Statistical Analysis

**Figure 2:**
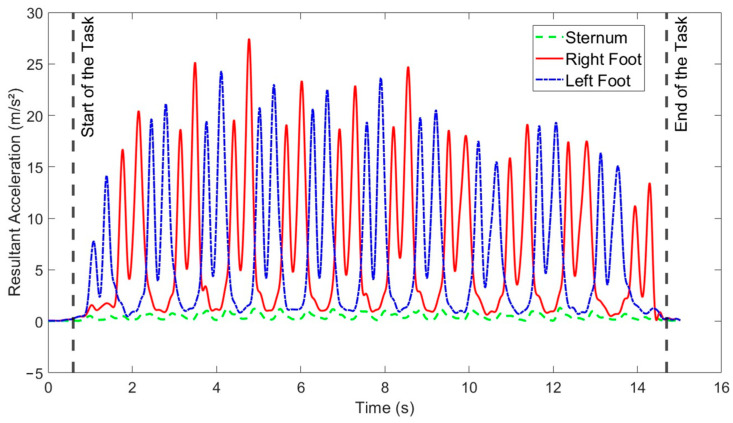
Illustration of the final plot of the resultant acceleration for the three IMU sensors after identifying the start and end of the 10MWT for a sample participant.

We created a custom MATLAB R2021B (MathWorks, Natick, MA, USA) code tailored for the analysis of the data collected. The data, which consisted solely of segment linear acceleration, underwent initial preprocessing and extraction using Xsens software (MVN Analyze^®^ 2021.0.1, Xsens, Enschede, The Netherlands). These data were employed to measure the duration of three specific tasks: the TUG test, STS task, and 10MWT. To accomplish this, we scrutinized the acceleration signals generated by the sensors. We identified the task start time by monitoring either the feet or sternum signals, and when these signals exceeded twice the standard deviation of the initial 30-data point window (equivalent to 0.3 s), we marked it as the task’s commencement. Subsequently, we applied a sliding window with a 30% overlap across the entire signal, and once it reached the same standard deviation threshold (as the initial window), we pinpointed the first data point in the window as the task’s end time. To ensure the reliability of this approach for signal analysis in each task, we conducted a visual inspection of the final plot for the selected points associated with each task. In cases where the selected points were deemed incorrect, we manually identified the correct start and end points for the tasks. Figure 2 illustrates a sample of the final plots displaying these selected points for the participants. Additionally, we determined the number of steps by counting the peaks present in the resultant acceleration signals [42,43].

During the balance tests, we recorded data over a duration of 30 s while the participants aimed to maintain as little movement as possible. To analyze these data, we computed the root mean square (RMS) of the resultant acceleration signals from four sensors: the sternum, pelvis, right thigh, and left thigh. We opted not to include the other four sensors located on the shanks and feet in the analysis for these tests. This decision was based on our initial analysis of three participants, which revealed minimal motion in the feet and sensors on the shanks. It was reasonable to anticipate more noticeable motion, particularly swinging, in the upper body. Subsequently, we calculated a total of 15 parameters encompassing a range of general kinematic variables and factors to quantify motion across the five tasks. These parameters encompassed the following:

1. TUG: Total Time (s), Steps toward Cone, and Steps toward Chair
2. STS: Total Time (s)
3. Balance Test (open eyes): Thorax Linear Acc (m/s^2^), Pelvic Linear Acc (m/s^2^), Right Thigh Linear Acc (m/s^2^), and Left Thigh Linear Acc (m/s^2^)
4. Balance Test (closed eyes): Thorax Linear Acc (m/s^2^), Pelvic Linear Acc (m/s^2^), Right Thigh Linear Acc (m/s^2^), and Left Thigh Linear Acc (m/s^2^)
5. 10MWT: Walk Time (s), Step, and Cadence (step/min)

It is important to note that all reported accelerations were derived from the RMS of the resultant linear acceleration specific to the relevant signal and sensor.

For data analysis, we employed a repeated measure ANOVA, constructing the model effects for each of the 15 factors under scrutiny using the independent factors of group (comprising the stroke and control participants) and CL (encompassing single and dual-tasks), along with their interaction. Prior to the analysis, we ensured that the prerequisites for repeated measures ANOVA were met, including confirming a normal distribution of the continuous dependent variable and the absence of outliers in any repeated measurements. Additionally, we calculated effect sizes (partial eta-squared: η^2^) for each analysis, categorizing them as low when η^2^ < 0.06, medium when 0.06 < η^2^ < 0.14, and large when η^2^ > 0.14 [44]. Throughout all analyses, a significance level of 0.05 was maintained, and the statistical software JMP Pro 16 (SAS Institute, Cary, NC, USA) was used for these computations. Lastly, post hoc analysis was performed on the interaction effect of group (SS or HI) and CL (single task or dual task) to assess significantly different levels.

## 3. Results

The main and two-factor interaction effect of group (stroke vs. control) and CL (single task vs. dual-task) are summarized in Table 2. A significant main effect of group was found for total TUG time, TUG steps toward cone and chair, balance eyes open thorax acceleration, balance eyes closed thorax acceleration, and 10MWT time and steps. The SS took longer, had more steps, and had more thorax sway compared to the controls across the tasks. There was also a significant main effect of CL on most balance sway measures, 10MWT time/steps/cadence, and total TUG time, indicating that dual tasking increased sway and gait alterations. A significant group × CL interaction was found only in right thigh linear acceleration (*p*-value = 0.021), suggesting that the effect of the dual task was significantly larger on the stroke group compared to the control group. However, for the rest of the measures, both groups were similarly affected by dual tasking.

Table 3 shows the means and standard deviations of key outcome measures for each group in single- and dual-task conditions. Total TUG time increased from single to dual tasks for both groups but was longer for stroke survivors (SS), especially in the dual task (18.44 s) compared to the controls (14.8 s). SS also had more thorax sway and slower 10MWT time in the dual task versus the single task. The lack of Group × CL interactions confirms that dual tasking impacted both groups to a similar degree across the clinical tests.

**Figure 3:**
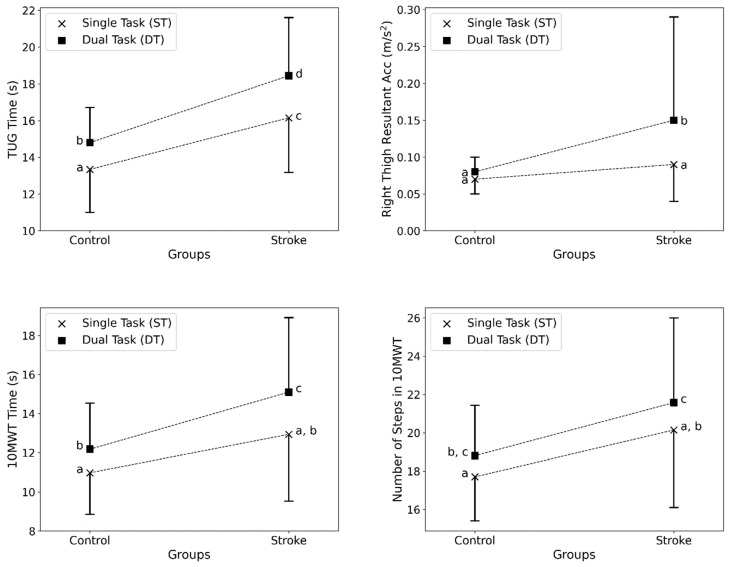
Comparison of key outcome measures between Stroke Survivors and healthy controls in single- and dual-task conditions. (**Top**-**left**) Total TUG time (s), (**top**-**right**) right thigh RMS acceleration (m/s^2^) during balance testing, (**bottom**-**left**) 10MWT time (s), (**bottom**-**right**) and number of steps during the 10MWT. Error bars indicate the standard deviation. (note: The points with no common lowercase letter labels were significantly different (*p*-value < 0.05) in the post-hoc analysis.)

Figure 3 visually illustrates some of the key findings summarized in Table 2 and Table 3. The top left plot shows that the total TUG time was longer for the SS compared to the controls, especially during dual tasking. The top right plot displays greater right thigh sway during balance testing in SS versus the controls when dual tasking. The bottom left and right plots exhibit slower 10MWT times and more steps for SS across conditions. The error bars represent the standard deviations, further demonstrating the larger variability observed in SS. Overall, the figure highlights the decrements in performance experienced by SS, particularly under dual-task conditions across the clinical tests. The larger error bars signify increased heterogeneity in the stroke group.

## 4. Discussion

This study aimed to investigate the impact of stroke and concurrent motor–cognitive dual tasking on a battery of common clinical assessments, which included the TUG test, STS task, balance assessment, and the 10MWT. Our key findings revealed that SS exhibited diminished performance, characterized by slower TUG times, increased sway during balance assessments, reduced gait speeds in the 10MWT, and an increased number of steps taken when compared to their healthy counterparts. Notably, the introduction of a cognitive dual task further exacerbated these impairments, leading to significant increases in TUG times, enhanced thorax sway during balance evaluations, and prolonged 10MWT completion times, particularly among SS. Interestingly, while these effects were pronounced, the lack of a significant interaction between the group and dual-tasks for most test outcomes (with the exception of right thigh linear acceleration) suggested that both stroke and control groups were similarly impacted by the dual tasking condition.

Earlier studies investigated the impact of stroke during the TUG test, focusing on the walking and turning performance of patients [45,46,47,48,49]. We observed very few studies that examine the effects of stroke and dual-tasks and even fewer that examined the interplay between them [29,50,51,52,53]. Nevertheless, the utility of dual-task TUG assessments has been established in identifying individuals at risk of falls in community settings [54,55]. Similarly, dual-task paradigms have been implemented in rehabilitation and training to improve performance [9,34,56]. Meanwhile, wearable sensors have demonstrated benefits in accurately detecting instances over spatial and temporal dimensions due to their high sampling frequency (e.g., up to 100 Hz) in recording the movement of body regions [57,58,59]. The novelty of this study lies in the implementation of both dual tasking and motion analysis by instruments in classic clinical tests to investigate impacts on the stroke population. Specifically, as in our earlier studies [46,60], by using wearable sensors, we were able to detect specific events at the start/end of the tests, which may be otherwise missed when using traditional methods (e.g., a stopwatch controlled by a clinician). The findings of our study show several significant differences in the outcomes of such tests (Table 3), indicating that incorporating dual tasks and motion analysis can be beneficial in precisely detecting affected functions in SS.

The outcomes of our investigation indicate that the primary impact of group (stroke vs. control) on the total TUG time, as well as the number of steps taken toward the cone and the chair, yielded statistically significant results (all *p*-values ≤ 0.005). These findings indicated that stroke patients, in comparison to the control group, exhibited a prolonged TUG duration and took more steps during the TUG task (as summarized in Table 2). However, when investigating the influence of the dual-task condition on these parameters, it is worth noting that the effect on the number of steps toward the chair did not reach statistical significance. To delve deeper into this observation, we can refer to the mean values (standard deviations) presented in Table 3. Here, we observe a slight increase in the number of steps toward the cone for both groups under dual-task conditions, with a 2% increase in the control group and a 5% increase in the stroke group. Conversely, a more substantial increase in the number of steps toward the chair (while returning after completing the turn around the cone) was evident in both groups, with a 7% increase in the control group and an 11% increase in the stroke group. This phenomenon may be attributed to the vestibular stimulation experienced upon turning around the cone, potentially impacting the participants’ balance, as suggested by prior research [45,61]. The addition of CL further compelled the subjects to take extra steps to maintain their balance while walking in this challenging dual-task scenario.

The lack of significant group or CL effects on STS performance could indicate that this test may not be sensitive enough to detect deficits in transitional movements post-stroke. STS relies heavily on leg strength and may not tax coordination or dynamic balance abilities that have been affected by stroke. In contrast, balance control and gait likely involve more complex motor planning and control and are more reliant on the integration of multiple sensorimotor systems impacted by stroke. Dual task costs were more apparent in these tests, suggesting that the division of attention resources exacerbated subtle postural control and locomotor deficits in SS (Figure 3). Damage to frontal lobe networks and reduced cognitive capacity may limit the ability to efficiently allocate attention when concurrently performing a cognitive task. Balance and gait require cognitive input for spatial orientation, planning motor sequences, monitoring execution. Thus, competing task demands appear to disproportionately affect stroke patients, revealing impairments not evident during single clinical tests. Further research is needed to elucidate the mechanisms underlying exaggerated cognitive–motor interference post stroke across different assessments. The findings support the value of dual-task testing to enhance sensitivity in detecting mobility limitations in complex real-world situations.

The mechanisms underlying exaggerated dual-task effects post-stroke are complex but likely involve divided attention demands exceeding limited cognitive resource capacity, as well as deficits in allocating attention between tasks. Damage to frontal lobe networks may disrupt the ability to efficiently switch between and coordinate cognitive-motor performance. Competition for resources appears greater when tasks require similar neural processing domains or when complex motor skills like walking are involved [62,63]. Interventions targeting dual-task abilities, such as virtual reality training integrating cognitive challenges during gait, could be a promising approach for improving mobility and reducing fall risk in SS similar to other parallel areas such as people with multiple sclerosis and Parkinson’s disease [64,65].

Our study represents an initial exploration into the utilization of dual-task clinical tests, focusing on how these two factors influence test outcomes. We expect that conducting assessments by incorporating dual-tasks and motion analysis can lead to more precise assessments of patients. As a natural progression of this research, a further examination could delve into how dual-task clinical tests might play a pivotal role in distinguishing between stroke patients across various dimensions. These dimensions encompass evaluating fall risk, enhancing rehabilitation strategies, and addressing other critical facets of stroke management. Consequently, our study serves as a foundational framework for future investigations aiming to shed light on the potential advantages of integrating dual-task assessments into the clinical care of stroke patients. Our study also highlights the potential of dual-task testing in revealing underlying mobility deficits and underscores the value of incorporating dual-task paradigms into stroke evaluations and targeted interventions, ultimately optimizing rehabilitation, and promoting safe community ambulation. Further research endeavors should delve into the individual factors that mediate cognitive-motor interference, thereby enabling more personalized and effective care.

Several limitations warrant acknowledgment in this study. Firstly, the relatively modest sample size employed may limit the generalizability of our findings, and the study did not consider the potential impact of heterogeneity in stroke location, severity, and cognitive profiles within the stroke survivor population. Additionally, our results may not be fully applicable to SS with lower levels of functioning, as the study participants were required to independently complete a 10 m walk. To enhance the robustness of future investigations, it is advisable to conduct research with larger and more diverse samples, thereby accommodating the inherent variability present in the stroke population. Future studies may also consider conducting short- and long-term studies that compare different patient groups using the methods demonstrated in this study. Furthermore, in this study, wearable sensors were exclusively utilized for balance testing. To bolster the depth of future research, the integration of advanced technology such as inertial sensors for the objective quantification of a wider range of clinical assessments is recommended. This would not only expand the scope of data collection but also provide a more comprehensive understanding of the multifaceted impact of stroke and dual tasking on motor-cognitive functions.

## 5. Conclusions

This study provides evidence that SS exhibit poorer performance on common clinical tests of mobility including the TUG test, STS task, balance tests, and 10MWT compared to HCs. Particularly, during single tasks, SS performed the TUG test and 10MWT at a slower pace, with it taking them ~2.8 s and 1.96 s more than the HC, respectively. SS also demonstrated 0.02 m/s^2^ more sway in the eyes open balance test. The addition of a concurrent cognitive task during testing further revealed disproportionate dual-task costs in the stroke group across the outcome measures. Specifically, dual tasking in SS increased TUG and 10MWT time by ~14% and 17%, with thorax sway increasing by ~64% during the balance test compared to the HC. The findings support incorporating dual-task paradigms into clinical evaluation post stroke, as this approach may better detect underlying mobility impairments not fully captured through single standardized tests alone. Dual task testing can help identify SS prone to falls who would benefit from comprehensive assessments and tailored interventions targeting identified cognitive-motor deficits. Future research with larger, more diverse stroke samples is warranted. Advances in sensor technologies should also be leveraged to enable the more objective quantification of standardized clinical tests and dual-task performance. Overall, this study underscores the importance of considering the interplay between cognitive loading and motor function when assessing and treating mobility challenges in stroke rehabilitation. Our findings encourage clinicians to integrate dual-task testing to optimize evaluation sensitivity and personalize interventions to improve safe mobility, reduce fall risk, and enhance participation for SS.

## Figures and Tables

**Table 1:** Demographic data of study participants categorized into groups of stroke survivors and healthy controls.

Parameters	Stroke Survivors (N = 21)	Healthy Controls (N = 20)	*p*-Value
Gender	11 males 10 females	8 males 12 females	-
Age (year)	66 (10)	60 (8)	0.053
Height (cm)	173.8 (8.4)	172.6 (9.6)	0.669
Weight (kg)	86.3 (14.7)	81.8 (18.2)	0.39
BMI (kg/s^2^)	28.5 (4)	27.2 (3.9)	0.288

**Table 2:** Main and interaction effects of the group (stroke and control) and cognitive load (single-task and dual-task) on the measures from the five tests.

Measures	Group	η2	CL	η^2^	Group × CL	η^2^
**TUG Test:**						
Total Time (s)	**<0.001**	0.244 (L)	**<0.001**	0.085 (M)	0.124	0.004 (S)
Steps toward Cone	**0.002**	0.202 (L)	0.113	0.005 (S)	0.371	0.001 (S)
Steps toward Chair	**0.005**	0.170 (L)	**0.022**	0.018 (S)	0.440	0.001 (S)
**Sit to Stand (x5) Test:**						
Time total (s)	0.289	0.019 (S)	0.150	0.002 (S)	0.215	0.008 (S)
**Balance Test (open eyes):**						
Thorax Linear Acc (m/s^2^)	**0.035**	0.022 (S)	**<0.001**	0.110 (M)	0.262	0.006 (S)
Pelvic Linear Acc (m/s^2^)	0.460	0.001 (S)	**<0.001**	0.108 (M)	0.118	<0.001 (S)
Right Thigh Linear Acc (m/s^2^)	**0.015**	0.024 (S)	**<0.001**	0.060 (M)	**0.021**	0.002 (S)
Left Thigh Linear Acc (m/s^2^)	**0.041**	0.030 (S)	**0.020**	0.062 (M)	0.711	<0.001 (S)
**Balance Test (closed eyes):**						
Thorax Linear Acc (m/s^2^)	**0.010**	0.111 (M)	**0.015**	0.021 (S)	0.376	0.007 (S)
Pelvic Linear Acc (m/s^2^)	0.251	0.036 (S)	**0.001**	0.004 (S)	0.861	0.000 (S)
Right Thigh Linear Acc (m/s^2^)	**0.021**	0.083 (M)	0.133	0.007 (S)	0.534	0.002 (S)
Left Thigh Linear Acc (m/s^2^)	**0.031**	0.081 (M)	0.277	0.006 (S)	0.396	0.002 (S)
**10-Meter Walk Test (10MWT):**						
Walk Time (s)	**0.010**	0.135 (M)	**<0.001**	0.064 (M)	0.257	0.005 (S)
Step	**0.019**	0.124 (M)	**<0.001**	0.029 (S)	0.504	<0.001 (S)
Cadence (step/min)	0.184	0.036 (S)	**<0.001**	0.070 (M)	0.152	0.007 (S)

Note: The partial effect size (η^2^) of the relevant effect analysis is reported in the right column. CL: cognitive load. The values in bold indicate statistical significance.

**Table 3:** Mean (SD) values of key outcome measures of the five tests for each group in the single-task (ST) and dual-task (DT) conditions.

Measures	Control (N = 20)		Stroke (N = 21)	
	Control ST	Control DT	Stroke ST	Stroke DT
**TUG Test:**				
Time total (s)	13.34 (2.34)	14.8 (1.91)	16.15 (2.97)	18.44 (3.17)
Steps toward Cone	6.05 (1)	6.15 (1.04)	7.19 (1.54)	7.57 (1.54)
Steps toward Chair	4.8 (0.83)	5.15 (1.04)	6.05 (1.56)	6.71 (2.55)
**Sit to Stand (x5) Test:**				
Time total (s)	17.81 (3.23)	19.06 (4.25)	20.04 (6.59)	20.6 (6.83)
**Balance Test (open eyes):**				
Thorax Linear Acc (m/s^2^)	0.08 (0.02)	0.14 (0.13)	0.1 (0.04)	0.19 (0.17)
Pelvic Linear Acc (m/s^2^)	0.05 (0.01)	0.05 (0.02)	0.05 (0.01)	0.06 (0.01)
Right Thigh Linear Acc (m/s^2^)	0.07 (0.02)	0.08 (0.02)	0.09 (0.05)	0.15 (0.14)
Left Thigh Linear Acc (m/s^2^)	0.07 (0.02)	0.1 (0.06)	0.11 (0.1)	0.18 (0.15)
**Balance Test (closed eyes):**				
Thorax Linear Acc (m/s^2^)	0.09 (0.03)	0.1 (0.03)	0.15 (0.12)	0.22 (0.23)
Pelvic Linear Acc (m/s^2^)	0.05 (0.02)	0.05 (0.02)	0.06 (0.02)	0.06 (0.02)
Right Thigh Linear Acc (m/s^2^)	0.08 (0.03)	0.09 (0.04)	0.14 (0.11)	0.17 (0.21)
Left Thigh Linear Acc (m/s^2^)	0.08 (0.04)	0.09 (0.05)	0.15 (0.12)	0.18 (0.22)
**10-Meter Walk Test (10MWT):**				
Walk Time (s)	10.98 (2.12)	12.18 (2.36)	12.94 (3.41)	15.1 (3.81)
Step	17.7 (2.3)	18.8 (2.63)	20.14 (4.04)	21.57 (4.42)
Cadence (step/min)	98.05 (8.87)	93.83 (9.1)	95.58 (13.23)	87.5 (12.81)

## Data Availability

The raw data supporting the conclusions of this article will be made available by the corresponding authors upon reasonable request.
